# Eco-friendly synthesis of coumarins using lemon juice as a natural catalyst and application as disperse dyes on polyester fabric

**DOI:** 10.1038/s41598-026-53238-4

**Published:** 2026-05-26

**Authors:** Ali A. Ali, Sawsan A. Fouad, Anhar Abdel-Aziem

**Affiliations:** 1 Department of Chemistry, Faculty of Science (boys), Al-Azhar University, Nasr City, 11884 Cairo Egypt; 2 Chemistry Department, Faculty of Science (Girls), Al-Azhar University, Nasr City, Cairo, 11754 Egypt

**Keywords:** Green synthesis, Natural catalyst, Coumarins, Polyester, Azodyes, Chemistry, Materials science

## Abstract

An efficient, low-cost, and eco-friendly protocol has been carried out for the synthesis of two coumarin derivatives using lemon juice as an eco-friendly acidic catalyst. The chemical structures of these derivatives were characterized using melting points as well as spectral data (IR, NMR, and MS). By optimizing dyeing factors like time, temperature, and pH, the study’s main goal was to ascertain how dispersion dyes **10** and **7** react when dyeing polyester fabrics. The colored polyester samples varied in color from light brown to yellowish brown to reddish brown to dark brown, depending on the coupler moieties at a depth of 5%. To achieve excellent colour strength in value (K/S = 21.7), the ideal dyeing conditions for dye **10** were 30 min, pH of 2, and 130 °C at shade 5%. While the ideal dyeing conditions for disperse dyes dye **7** (K/S = 20.8) were 30 min, pH of 4, and 130 °C at a shade of 5%. When everything is considered, synthetic dyes appear to be good choices for adding a variety of colors to polyester textiles.

**Supplementary Information:**

The online version contains supplementary material available at 10.1038/s41598-026-53238-4.

## Introduction

Growing environmental concerns associated with conventional chemical processes have driven increasing interest in sustainable and eco-friendly methodologies. Traditional organic synthesis often depends on hazardous solvents and corrosive mineral acids, generating significant environmental impact^1–12^. In response to these challenges, the concept of green chemistry was introduced by Paul Anastas and John Warner in 1991, emphasizing waste minimization, mild reaction conditions, high yields, and the use of renewable resources^13–17^. Among various green strategies, natural catalysts derived from renewable resources have emerged as cost-effective and environmentally benign alternatives^18[19][20][21]^. Fruit extracts, particularly lemon juice, contain organic acids such as citric acid, which can promote condensation reactions under mild conditions, offering a biodegradable and eco-friendly substitute for conventional mineral acids^22^. Recently, natural dye sources and sustainable coloration approaches have gained increasing attention due to their environmental compatibility and biodegradability^23,24^.

Due to their numerous applications in a variety of industries, azo dyes and pigments are among the most pertinent types of organic colorants^25,26^. The textile industry benefits from azo dyes’ well-known nitrogen-nitrogen double bond in several ways^27^. Azo dyes have long been used to impart color to a wide range of things, such as food, paper, clothes, cosmetics, drugs, and dye-sensitized solar cells^28^, Antioxidant-containing working fluids for solar applications at high temperatures^29^, For printing on polyester and beyond, digital transfer printing ink with colored pigments^30,31^. The dye’s color is affected by the conjugation system, auxochrome group, chromophore, and wavelength absorption in the visible portion of the dye molecule^32^. The title chemical is usually created by combining a diazonium salt with one or more electron-rich nucleophile chains^33,34^. The diazotization technique is used to first transform the aromatic or heterocyclic amine into a diazonium salt^35^. The conventional diazotization process produces a diazonium product that interacts at low temperatures with various diazo-coupling nucleophile components such as phenol, naphthol, or amine when salts and acids are present^36^.

Coumarin is an oxygen-containing heterocyclic compound characterized by a benzopyrone framework with an extended π-conjugated system, which enables efficient absorption in the ultraviolet and visible regions. Owing to its rigid planar structure and strong electron delocalization, coumarin derivatives exhibit high molar absorptivity, intense coloration, and favorable photophysical properties^37,38^. These features, combined with good photostability and strong affinity toward synthetic fibers, particularly polyester, make coumarin-based compounds promising candidates for high-performance disperse dyes in textile applications^39,40^. Furthermore, structural modification through the incorporation of heterocyclic moieties such as the thiazole unit can further improve chromophoric properties, enhancing dyeing efficiency and color intensity^41^. Despite extensive studies on coumarin-based dyes and sustainable synthetic methodologies, the integration of natural-catalyst-assisted synthesis with direct textile application on polyester fabrics remains insufficiently explored. In continuation of our ongoing research program on the development of green and sustainable synthetic methodologies for heterocyclic compounds using environmentally benign catalytic systems^42–44^, the present work further advances this direction. In this work, lemon juice is introduced as an efficient, eco-friendly, natural acidic catalyst for the preparation of two coumarin derivatives, which are further explored for their application as disperse dyes for polyester fabrics, highlighting their potential for sustainable large-scale textile applications. The chemical structures of two recently synthesized, environmentally friendly azo dye molecules with a high yield (90%) based on coumarins have been clarified using spectroscopic, elemental, and UV-Vis techniques. The color position in colorimetric coordinates (CIEL*a*b*), color strength properties (K/S), auxochrome effect, and color intensity qualities will all be assessed by applying the generated dyestuffs to polyester fabrics.

## Experimental

### Materials and instrumentation

All chemical reagents and solvents used in the experiments were purchased from Sigma-Aldrich, Germany, and used without further purification. The melting points of the prepared Schiff bases were measured on an electrothermal apparatus. Infrared (IR) spectra were detected on a Nicolet is 10 FTIR instrument within the wavenumber range of 4000–400 cm⁻¹ by the KBr disk technique. A Jeol spectrometer (Japan) operating at 500/125 MHz was utilized to measure ¹H and ¹³C (NMR) in DMSO-d_6_ as solvent and tetramethylsilane (TMS) as the internal standard, and chemical shifts are expressed in δ. The following symbols were used to describe the hydrogen coupling patterns (br. = broad, s = singlet, d = doublet, t = triplet, q = quartet, and m = multiplet) and coupling constant (*J*) in Hertz (Hz). Using infrared dyeing equipment from AHIBA laboratories, the dyeing procedure was completed. The colorimetric characteristics of the colored fabric were ascertained using a Data-Color 850. El-Nahawy Textile Company, an Egyptian company, provided (140 gm/m^2^, plain weave, 66-inch width, 0.39 mm/cm thickness, 48 * 150 denier yarn, and 110 * 80 warp threads in the weft.

### Green Synthesis

#### Preparation of lemon juice as a biocatalyst

Fresh lemon fruit was washed, cut, and manually squeezed. The juice obtained was filtered using filter paper to remove all solid materials and use it as a clear juice in the reaction.

#### Synthesis of 3-acetyl-6-bromo-chromen-2-one (3)

This compound was prepared by grinding 5-bromosalicylaldehyde (**1)** (2.01 g, 0.01 mol) with ethyl acetoacetate (**2)** (1.30 ml, 0.01 mol) with a pestle in an open mortar at room temperature in the presence of three drops of piperidine. After 10 min of grinding, the solid was washed with ethanol, affording **3** with mp = 220.

### Synthesis of 6-bromo-3-(2-bromo-acetyl)-chromen-2-one (4)

To a stirred solution of ketone **3** (0.01 mol) in 10 ml of chloroform, a solution of bromine (0.01 mol) in chloroform was added dropwise at room temperature. After one hour, the crude solid was added to ice, filtered, washed several times with water, and recrystallized from acetic acid to give compound **4** as a pale-yellow powder with mp = 178 ^◦^C (lit. 177–179^◦^C).

#### Synthesis of 6-bromo-3-(2-hydrazino-thiazol-4-yl)-chromen-2-one (6)

6-Bromo-3-(2-bromoacetyl)−2*H*-chromen-2-one (**4**) (0.01 mol) was taken in a round bottom flask. Then thiosemicarbazide (**5**) (0.01 mol) and 3 mL of lemon juice were added, and the mixture was refluxed. After 5 min, the yellow precipitate that formed was collected, dried, and recrystallized from DMF, yielding the desired product **6**. M.*p* = 291 °C (lit. 292 °C).

#### Synthesis of (*Z*)−6-bromo-3-(2-(2-(1-(6-bromo-2-oxo-2*H*-chromen-3-yl)ethylidene)hydrazinyl)thiazol-4-yl)−2*H*-chromen-2-one (7)

##### Method A

In a round-bottom flask, a mixture of 3-acetyl-6-bromo-2*H*-chromen-2-one **(3)** (0.01 mol) and 6-bromo-3-(2-hydrazino-thiazol-4-yl)-chromen-2-one (**6**) (0.01 mol) in 3 ml lemon juice was heated under reflux. After 10 min, the yellow precipitate formed was filtered, washed with water to remove the acid, dried, and recrystallized from DMF to afford compound **7** in 92% yield.

##### Method B

An equal amount of 3-acetyl-6-bromo-2 H-chromen-2-one (3), 6-bromo-3-(2-bromoacetyl)−2* H*-chromen-2-one (**4**) (0.01 mol), and thiosemicarbazide (**5**) (0.01 mol, each), was taken in a round-bottom flask. After adding 3mL lemon juice, the mixture was heated under reflux. The solid formed after 10 min. was then filtered, washed with water, dried, and recrystallized from DMF to afford **7** in 90% yield.

Brown powder, m.p: 260 − 62 ℃; IR (KBr) cm^− 1^: 3404 (N-H), 3095,2923 (CH), 1734,1678 (2 C = O), 1604 (C = N), 1550 (C = C); ^1^H NMR: (500 MHz, DMSO-*d*_6_, δ, ppm): 2.55 (s, CH_3_), 7.40 (d, 3 H, *J* = 8.55 Hz), 7.75 (s, 1H, NH), 7.84 (d, 2 H, *J* = 8.55 Hz), 8.17 (s, 2 H), 8.56 (s, 2 H). Anal. calcd. for C_23_H_13_Br_2_N_3_O_4_S (587): C, 47.04; H, 2.23; Br, 27.21; N, 7.16; S, 5.46. Found: C, 47.10; H, 2.29; Br, 27.12; N, 7.11; S, 5.51.

#### Synthesis of 6-bromo-3-{1-[(4-phenyl-5-phenylazo-thiazol-2-yl)-hydrazono]-ethyl}-chromen-2-one (10)

A mixture of thiosemicarbazone **8** and hydrazonoyl halides **9** (0.01 mol of each) were grinded in a mortar with a pestle at room temperature in the presence of three drops of trimethylamine. After 10 min, the colored solid was collected, washed with ethanol, and recrystallized from DMF, affording **10** in 90% yields.

Orange powder; m.p: 225 − 26 ℃; IR (KBr) cm^− 1^: 3428 (N-H), 3067, 2924 (CH), 1735 (C = O), 1644 (C = N), 1599 (C = C); ^1^H NMR: (400 MHz, DMSO-*d*_6_) δ = 2.38 (s, 3 H), 7.42 (t, 2 H, *J* = 8.8 Hz), 7.58–7.63 (m, 5 H), 7.72, 1H, *J* = 8 Hz), 7.78, 7.81 (dd, 1H, *J* = 1.7 Hz, *J* = 2.4 Hz), 8.02 (d, 2 H, *J* = 9.1 Hz), 8.22–8.26 (m, 3 H), 8.30 (s, NH); ^13^C NMR (125 MHz, DMSO-*d*_6_ δ, ppm): 17.90 (CH_3_), 116.82, 118.75, 121.26, 123.05, 123.39, 127.37, 128.03, 129.27, 129.71, 130.78, 131.95, 134.61, 134.80, 135.35, 139.21, 140.94, 150.35, 151.79, 153.09, 158.93, 159.43 (Ar-C), 183.16 (C = O). Anal. calcd. for C_26_H_18_BrN_5_O_2_S (544.42): C, 57.36; H, 3.33; Br, 14.68; N, 12.86; S, 5.89. Found: C, 57.43; H, 3.26; Br, 14.75; N, 12.80; S, 5.97.

### After dye dispersions are made, the dyeing process

AHIBA lab process for color information. Polyester clothes were colored using infrared dyeing equipment. The rate and volume of dispersed dye uptake on hydrophobic fibers are influenced by a leveling and dispersing agent that increases the dyes’ aqueous solubility and affinity for the aqueous phase. In addition to the 25 mL dye bath supplied by an Egyptian chemical company that contains 2.0 mL/l of Kimi-leveling ES, a leveling-dispersing agent made from fatty acid ester, and two drops of dimethylformamide^45^. As mentioned earlier, the dispersion dye was made. The pH of the dye bath was elevated to 8 and 10 by sodium hydroxide and lowered to 2, 4, and 6 by acetic acid^45^. The polyester textiles are submerged in a dye bath that is heated at a rate of two degrees Celsius per minute. They reach temperatures in the range of 110 to 130 °C. Depending on the required temperature, the dye bath is kept at this high temperature for half an hour^46^.

A 1:20 liquor ratio was used to combine sodium hydroxide (2 g/l) with sodium hydrosulfite (2 g/l) in an aqueous solution. At 85 °C, the reduction-clearing process ran for 20 min. They were submerged in a separate bath containing one gram of acetic acid per liter for five minutes at 60 degrees Celsius to reduce their pH.

### Evaluation of color yield

The CIELAB color strength values (K/S), reflectance value, and color coordinates (L*, a*, b*, C*, and H^o^) of colored fabric samples were determined using a Data-Color 850 spectrophotometer^47^. The surface color strength of colored cloth samples was measured using the K/S value, and the color strength of the fabric was evaluated using the reflectance value at a maximum wavelength^48^. The effects of the various substituents on the dyeing behavior, color hue, and depth were examined using spectral data from the dyed materials. The Kubelka-Munk function f(R), which describes the light absorption “K” and scattering “S” of a particular sample, was used to analyze the optical characteristics of the materials. The coloring components significantly alter “K” absorption. Because the substrate’s influence on the scattering “S” is simultaneous, the reflectance (R) of a thick, opaque material with constant values of “K” and “S” can be found using Eq. (1), which is based on the wavelength of light, and the Kubelka-Munk theory^49,50^. The color difference (ΔE) of the dyed polyester fabric samples was determined using Eq. (2)^51^.

1 $$\:\mathbf{K}/\mathbf{S}=\frac{(1-\boldsymbol{R})2}{2\boldsymbol{R}}$$

2 $$\Delta\:E= [(\Delta \:L)^{2} + (\Delta\:b)^{2}+ (\Delta\:a)^{2}]^{1/2}$$

3 $$C^{*} = (a^{2} + b^{2})^{1/2}$$

4 $$H^{o} = tan^{--1} a/b$$

The ranges of lightness (L*) (0–100), hue angle (H^o^), and chroma (C*) are 0 to 360 degrees; the degrees of green (negative) and red (positive) are indicated by a*, and blue (negative) and yellow (positive) by b*. The dispersed colors of the polyester cloth all migrated in a yellowish direction, resulting in positive values on the yellow-blue axis, or b*^52,53^.

### Polyester fabric dyeing evaluation

In compliance with ISO 105-C10:C:2010, wash fastness was evaluated both before and after the reduction cleaning bath using a multifiber adjacent cloth at 60 °C for 30 min. The fastness ratings were recorded on a scale of 1 to 5. Light fastness: The AATCC TM16 method was used to rate the results on a scale of 1 to 8. Crock Fastness: Determined using a Crockmeter in compliance with ISO 105-X12:2016. Ratings were assigned on a scale of 1 to 5 ^**54**^.

## Results and discussion

### Chemistry

**Scheme 1:**
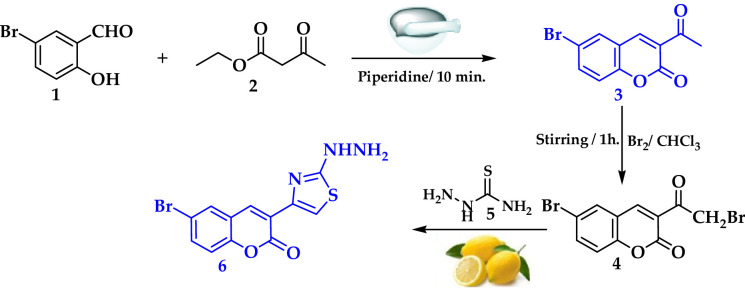
Synthesis of intermediates **3**, **4**, and **6**.

Building on our previous synthesis of coumarin derivatives via traditional stirring and reflux methods, we recognized several significant limitations.[55-58] These conventional approaches often suffered from extended reaction times, excessive energy consumption, and a reliance on hazardous, non-renewable organic solvents. [59-62] To align with the 12 Principles of Green Chemistry, this study introduces a sustainable, solvent-free approach. We utilize mechanochemical grinding combined with fresh lemon juice—a natural source of 5–7% citric acid—as a renewable, bio-based acid catalyst to improve efficiency and reduce environmental impact for developing more green protocols. In current research, 3-acetyl-6-bromo-2*H*-chromen-2-one **(3)** was prepared by simple grinding of 5-bromosalicylaldehyde (**1)** with ethyl acetoacetate (**2)** under Kneovenagel condensation conditions. Bromination of the acetyl **3** with Br_2_ in chloroform yielded 6-bromo-3-(2-bromo-acetyl)-chromen-2-one (**4**), which underwent Hantzsch thiazole synthesis via condensation with thiosemicarbazide (**5**), giving 6-bromo-3-(2-hydrazino-thiazol-4-yl)-chromen-2-one (**6**) as shown in Scheme 1.

**Scheme 2:**
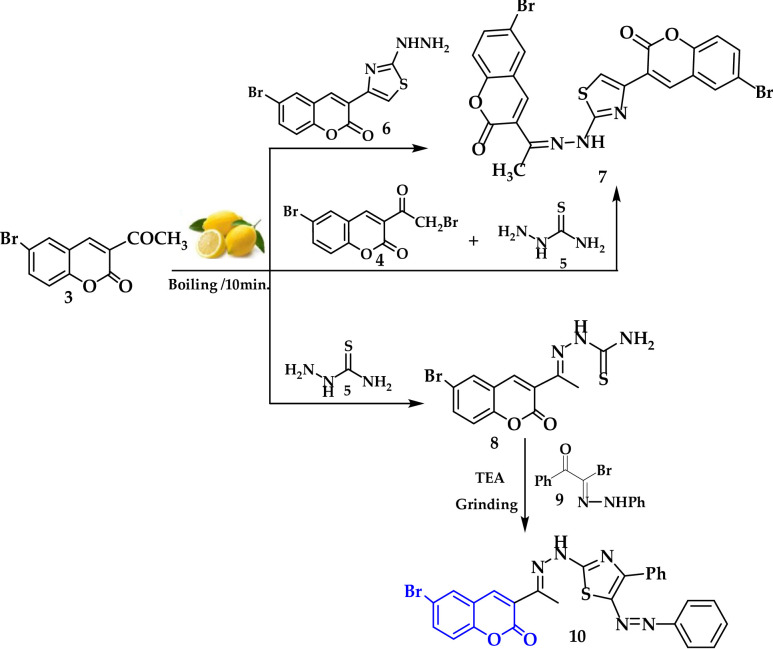
Lemon juice catalyzed the synthesis of coumarin bearing thiazole moiety **7** and **10**.

The desired 6-bromo-3-(2-(2-(1-(6-bromo-2-oxo-2*H*-chromen-3-yl)ethylidene) hydrazinyl)thiazol-4-yl)−2*H*-chromen-2-one (**7**) was produced by condensation reaction of 3-acetyl-6-bromo-2*H*-chromen-2-one **(3)** with the synthesized compounds hydrazinyl thiazole derivative **6** using lemon juice under boiling for 10 min (Scheme 2). Under the same reaction conditions, Schiff base **7** can be achieved by multicomponent one-pot condensation green Biginelli reaction of ketone **3**, thiosemicarbazide (**5**), and 6-bromo-3-(2-bromo-acetyl)-chromen-2-one (**4**). Fourier transform infrared, NMR, and MS spectral data were used for elucidation of structures. Compound **7** exhibited absorption peaks at 3404, 1734, and 1678 cm^− 1^, indicative of imino and two carbonyl groups. In the^1^ 1H NMR spectrum of compound **7**, singlet signals at δ 2.55 and 7.75 ppm were observed for methyl and NH protons, respectively.

Next, thiosemicarbazone **8** was produced via acid-catalyzed condensation reaction between ketone **3** and thiosemicarbazide (**5**) as earlier reported. It underwent Hantzsch thiazole synthesis upon grinding with 2-oxo-*N*−2-diphenylacetohydrazonoyl bromide (**9**), and three drops of triethylamine to produce the corresponding thiazole derivative **10** in good yield.

### Characterization of the synthesized dyes by UV-Vis

The visual absorption spectra of dyes **7** and **10** in three distinct solvents (DMSO, Aceton, and DMF) at a concentration of 5 × 10⁻³ mg/L are shown in Fig. 3 to investigate the impact of solvent polarity on their spectral properties. The spectral characteristics, such as full width at half maximum (FWHM), maximum absorption wavelength (λ_max_), and molar absorption coefficients (Ɛ), are summarized in Table 1. The Beer-Lambert rule (A = Ɛ ×C) was validated by the linear plots of absorbance (A) vs. concentration (C)^63^. To assess the impact of the solvent environment on the photophysical behavior of the examined dyes (dye **7** and dye **10**), the UV–Vis absorption spectra were recorded in three solvents of varying polarity: DMF, DMSO, and acetone. Variations in electronic structure and solute-solvent interactions are reflected in the spectral data, which show clear variances in band shapes and absorption maxima (λ_max_).

**Fig. 1:**
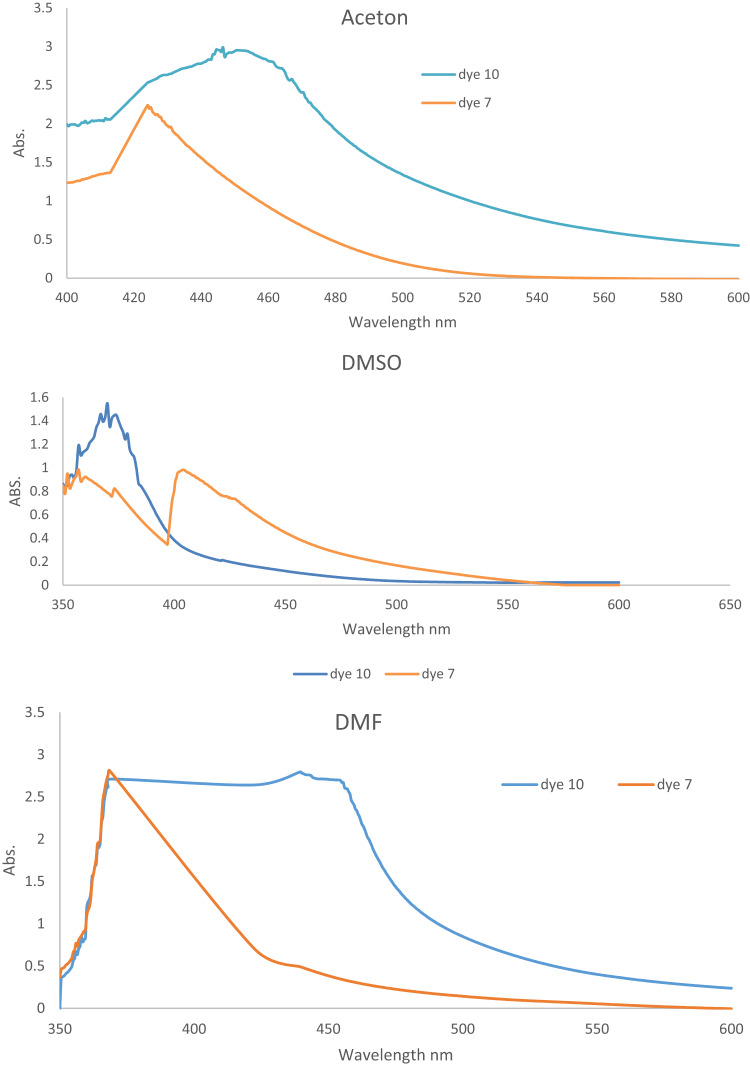
Displayed the effect of solvent polarity on the absorption spectra of dyes **7** and **10** in various solvents: Aceton, dimethyl sulfoxide (DMSO), and dimethylformamide (DMF).

In contrast to dye 7 (λmax = 375–430 nm), dye 10 regularly shows a bathochromic shift (λmax ≈ 445–455 nm), suggesting a longer π-conjugation system and higher intramolecular charge transfer (ICT) character A small red shift occurs as the polarity of the solvent is increased (from acetone to DMSO), especially for dye **7**, indicating that the excited state is more stable in polar media. A more intrinsically stable electronic structure is implied by Dye 10’s reduced sensitivity to solvent alterations. The molar absorption coefficients (Ɛ) range from ((1.87–3.5) × 10³ for dye **7**) and (2.64–3.3 × 10³) for dye **10**) L·mol⁻¹·cm⁻¹, with dye **10** often having a larger value than dye **7**. However, because of the high absorbance, readings obtained in DMF and acetone indicate apparent Ɛ, but DMSO offers more accurate data within the linear range of the Beer-Lambert Law. FWHM analysis reveals that dye **7** has smaller bands (35–60 nm), indicating more localized π→π* transitions, while dye **10** has larger absorption bands (~ 90 nm), consistent with ICT behavior and increased electronic delocalization (Fig. 1; Table 1).

**Table 1:** The dyes’ (7 and 10) photophysical characteristics in various solvents.

Solvent	Relative Polarity	Absorbance at maximum		FWHM		λ_max_(nm)		Ɛ (L.mol^− 1^ cm^− 1^)	
		Dye 7	Dye 10	Dye 7	Dye 10	Dye 7	Dye 10	Dye 7	Dye 10
**DMF**	**0.386**	**3**	**2.8**	**35**	**90**	375	**445**	**3.5** × **10**^**3**^	**3.05** × **10**^**3**^
**DMSO**	**0.444**	**1.2**	**1.5**	**60**	**90**	430	**455**	**1.87** × **10**^**3**^	**2.64** × **10**^**3**^
**Aceton**	**0.355**	**2.2**	**3**	**50**	**90**	420	**450**	**2.6** × **10**^**3**^	**3.3** × **10**^**3**^

### CLogP computation

The CLogP server in the Chem-Draw application was used to calculate the dyes’ ClogP (N-octanol/water partition coefficient) values. To disperse the color of polyester, the dyes must be split between two immiscible solvents (water and polyester fibers). Consequently, the dye will migrate more to the hydrophobic polyester fibers if it is less soluble in the dyebath and more hydrophobic. Therefore, the following section discusses ClogP values. In the aqueous dyebath, a lower ClogP usually denotes better dye solubility dispersion. The final K/S values are negatively correlated with the solubility of the dispersion dye^54^. Thus, dye 7 should exhibit greater K/S ratios than dye 10, which has the maximum solubility due to its lower ClogP value of 5.184, whereas dye 10 has the lowest solubility in an aqueous dye bath due to its higher ClogP value of 6.978. Therefore, even though dye 7 displayed higher ClogP values than dye 10, it’s likely that dye 10, which is the smallest, diffused more in the polyester polymer matrix and so revealed higher K/S values.

### Features of dye transfer in polyester textiles

#### Measurements for analysis and color strength (K/S)

**Fig. 2:**
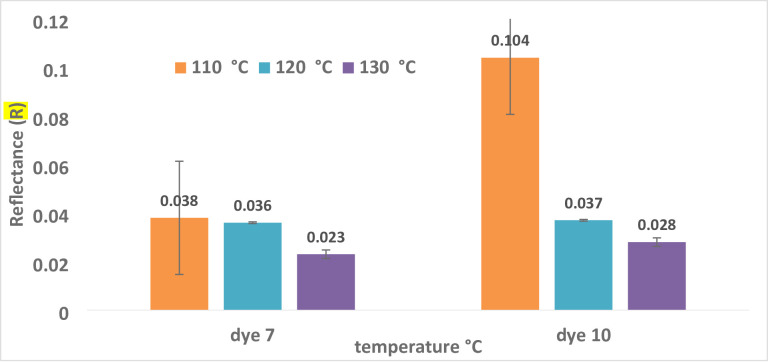
For dyes **7** and **10**, the relationship between temperature and reflectance R is as follows: time = 30 °C, pH = 4, shade = 5.

**Fig. 3:**
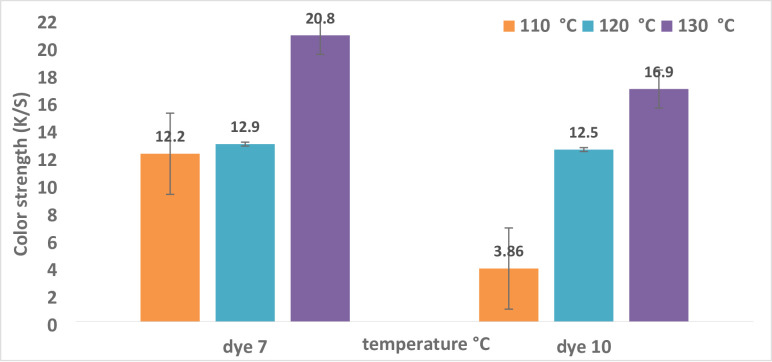
There is a correlation between temperature and color strength (K/S) for dyes **7** and **10** (time = 30 °C, pH = 4, shade = 5%).

**Fig. 4:**
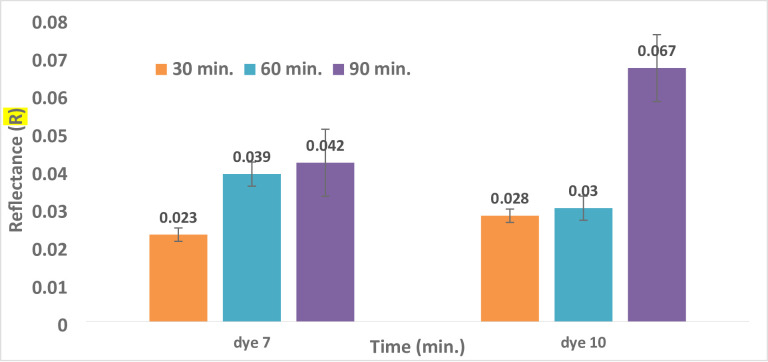
Time and Reflectance (R) relationship for dyes **7** and **10** at (temperature = 130 °C, pH = 4, and 5% shade).

**Fig. 5:**
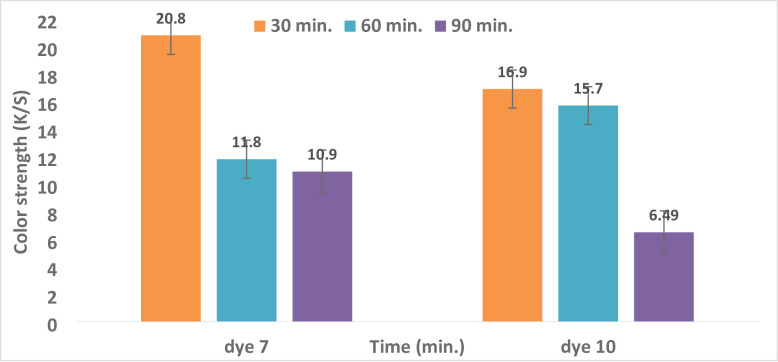
Time and color strength (K/S) relationship for dyes **7** and **10** at (temperature = 130 °C, pH = 4, and 5% shade).

**Fig. 6:**
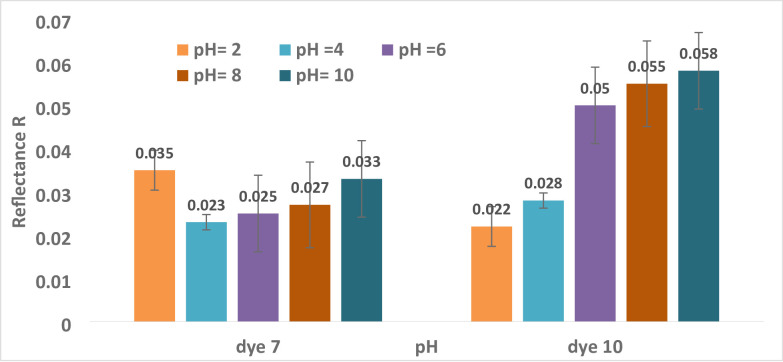
The relationship between pH and reflectance R for dyes **7** and **10** is as follows (time = 30 °C, Temp. = 130 °C, shadow = 5).

The K/S values at the wavelengths with the highest absorbance or lowest reflectance (380 and 390) are frequently used to characterize the behavior of dye deposition on textile surfaces. With positive values for a* and b*, respectively, the color hues of the dye changed to the yellowish direction on the yellow-blue axis and the reddish direction on the red-green axis for all colored fabrics **7** and **10** at various settings figures (1–6) and Tables 1, 2 and 3. The hue angle (H^o^) for each of these three artificial dyes shifts to a yellowish hue when it is positive and a reddish hue when it is negative^64,65^

The amount of visible light reflected by a colored cloth is indicated by its reflectance value. White fabric samples have a reflectance value of 100% since they reflect all light wavelengths, whereas black fabric absorbs all light wavelengths and has a reflectance value of zero^54,66^. Consequently, all other colors have reflectance values that fall between these two poles. The chemical structure and substituent of the dyes’ aromatic moiety created a variety of dyeing values, which affected the K/S values. Dye **10** yielded better results because it was a brighter hue than dye **7**.

#### Reflectance (R) and color strength (K/S) as a function of dyeing temperature

This technique used two dispersion dyes to color polyester samples at temperatures between 110 and 130 °C. The impact of temperature variations on the color strength (K/S) of polyester dyes is shown in Table 2; Fig. 2. At temperatures higher than their glass transition point (80 °C), polyester fibers necessitate the use of dispersion colors. This experiment began at 110 °C since that temperature is always selected over 80–100 °C, as was the case in earlier studies^ 66,67^. As shown in Fig. 2, the color strength of polyester fabric treated with a dye bath rises to equilibrium. The shifting proportion of the dye from the fabric to the dye bath may account for this alteration.

Figure 2 indicates that the ideal temperature for dyes **7** and **10** was 130 °C, with values of 20.8 and 16.9, respectively. This suggests that the limiting temperature of 130 °C, where equilibrium is reached, is the optimal temperature for dispersing dyes **7** and **10** in water with a dispersing agent present. This could be explained by the dye molecules’ high kinetic energy and rapid dispersion rate. Consequently, the optimal temperature for the application was found to be 130 °C^54^. If the temperature is greater than ideal, the dye may not transfer onto the cloth, reversing the dyeing process and reducing the K/S and fastness qualities^54^. Table 2; Fig. 3 illustrate how the value of reflection decreases with increasing temperature for dyes **7** and **10**.

**Table 2:** In dyes **7** and **10**, the measurements of L*, a*, b* E, H^o^, C*, R, and color strength are affected by the dyeing temperatures (pH = 4, 5% shade, and time = 30 min).

Dye	Temp. (°C)	Dyeing fiber’s color hues	L*	a*	b*	C*	H^o^	E	*R*	K/S
7	110		69.7	4.3	31.8	32.1	82.2	76.7	0.038	12.2
	120		69.5	5	35.7	36.1	82	76.5	0.036	12.9
	130		57.1	7.63	32.2	33.1	76.7	66	0.023	20.8
**10**	**110**		**74.7**	**2.4**	**33.3**	**33.4**	**85.9**	**81.8**	**0.104**	**3.86**
	**120**		**62.3**	**7.6**	**41.5**	**42.2**	**79.7**	**75.2**	**0.037**	**12.5**
	**130**		**53**	**8.12**	**36.8**	**37.6**	**77.5**	**75.2**	**0.028**	**16.9**

#### 3.4.3. Impact of Dyeing Time on Color Strength (K/S) and Reflectance (R)

The K/S values of polyester fabrics dyed for 30, 60, and 90 min are shown in Fig. 4. For different lengths of time, polyester was dyed at 130 °C in an acidic environment (pH 4 for dyes **7** and **10**). For dyes **7** and **10**, longer dyeing times result in a drop in color strength (K/S). After 30 min of dyeing, dyes **7** and **10** reached their maximum K/S values (20.8 and 16.4, respectively), as shown in Fig. 4; Table 3. In every dye scenario, the distribution of dye molecules between the fiber and the dye solution reached equilibrium after a variable amount of time. The requirement for equilibrium in the treatment bath and the potential for stripping from prolonged heating could account for this^47,65^. The reflectance values of all hues gradually increased over time for dyes **7** and **10**, as shown in Fig. 5; Table 3. The best time to dye polyester with colors **7** and **10** is 30 min, according to the investigation’s conclusions.

**Table 3:** The dyeing times of dyes **7** and **10** (temperature = 130 °C, pH = 4, and 5% shade) have an impact on color measurements, color strength (K/S), and reflectance percentage.

Dye	Time min	Color shades	L*	a*	b*	c*	H^o^	E	*R*	K/S
7	30		57.1	7.63	32.2	33.1	76.7	66	0.023	20.8
	60		56	13.1	26	24.1	63.4	61	0.039	11.8
	90		57.3	11.6	23.3	26	63.5	62.9	0.042	10.9
**10**	**30**		**53**	**8.12**	**36.8**	**37.6**	**77.5**	**65**	**0.028**	**16.9**
	**60**		**56**	**8.5**	**39**	**40**	**77.8**	**68.8**	**0.03**	**15.7**
	**90**		**66.3**	**7.72**	**35.7**	**36.5**	**77.8**	**75.7**	**0.067**	**6.49**

#### Dyeing pH has an impact on both Reflectance (R) and Color Strength (K/S)

**Fig. 7:**
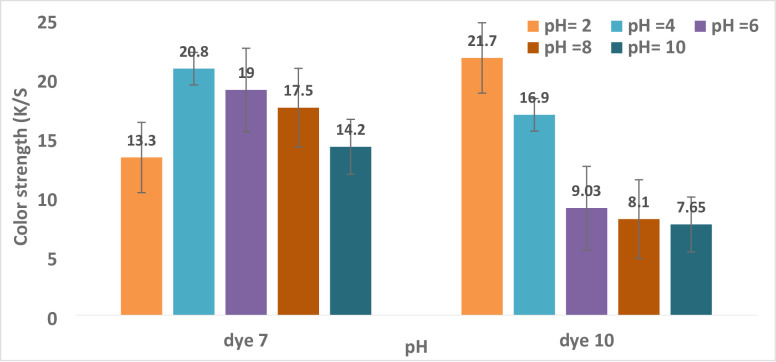
The relationship between pH and color strength (K/S for dyes **7** and **10** is as follows (time = 30 min., Temp. = 130 °C, shadow = 5).

However, an acidic pH is often used in polyester dyeing. The investigation’s findings show that dyeing polyester with dye **10** requires a pH of 2, while dyeing polyester with dye **7** requires a pH of 4, as shown in Table 4; Fig. 6. With each pH increase from 4 to 10, the color intensity decreased, and all hues displayed a logical order. The order of preference for dye **10** was 2 > 4>6 > 8>10 with values of (21.7,16.9, 9.03,8.1, and 7.65), respectively; for dye **7**, the order of preference was 4 > 6>8 > 10>2 with values of (20.8,19,17.5,14.2, and 13.3), respectively. Figure 7; Table 4 demonstrate how the reflectance values of all hues for dyes **7** and **10** progressively rose over pH. Changes in dye ionization, aggregation behavior, and dye–fiber interactions are the primary causes of pH’s impact on dyeing performance. Due to improved protonation, increased hydrophobicity, and a stronger affinity for polyester, Dye **10** shows a higher K/S at pH 2. Dye **7**, on the other hand, performs poorly at pH 2 because strong intermolecular hydrogen bonding causes huge aggregates to develop, which prevents diffusion into the fiber. Maximum dye uptake occurs at a pH of 4–6, which is the ideal compromise between dispersion stability and aggregation. Lower color strength results from less substantivity due to higher dye solubility at alkaline pH. ^68,69^[68, 69].

**Table 4:** The dyeing pH (temperature = 130 °C, 5% shade, and time = 30 min.) has an impact on the measurements of L*, a*, b*, E, H^o^, C*, R, and color strength in dyes **7** and **10**.

Dye	pH	Dyeing fiber’s color hues	L*	a*	b*	C*	H^o^	E	*R*	K/S
7	2		71.7	6.62	34.7	35.3	79.2	79.9	0.035	13.3
	4		57.1	7.63	32.2	33.1	76.7	66	0.023	20.8
	6		57.6	5.24	31.4	31.9	80.5	65.8	0.025	19
	8		59.9	5.8	33.4	33.9	80.1	68.8	0.027	17.5
	10		62.1	4.9	28	28.5	80.1	68.3	0.033	14.2
**10**	**2**		**42.9**	**6.75**	**29.6**	**30.3**	**77.2**	**52.5**	**0.022**	**21.7**
	**4**		**53**	**8.12**	**36.8**	**37.6**	**77.5**	**64.9**	**0.028**	**16.9**
	**6**		**58.6**	**7.4**	**33.3**	**34.1**	**77.6**	**67.7**	**0.05**	**9.03**
	**8**		**67.8**	**7.2**	**39**	**39.7**	**79.6**	**78.6**	**0.055**	**8.1**
	**10**		**63.8**	**11.9**	**34**	**36.1**	**70.6**	**73.3**	**0.058**	**7.65**

According to the results, the addition of the thiazole ring improves planarity and strengthens π–π interactions with the polyester fiber by increasing electron delocalization within the chromophoric system. Increased dye uptake is shown by reduced reflectance and increased color strength (K/S) values because of this structural alteration. Additionally, compared to more polar analogues, the thiazole unit’s presence lessens excessive aggregation, enhancing dye dispersion and homogeneity^70^. In conclusion, dye No. **7** has a higher K/S (20.8) than dye No. **10** (21.7). The color strength of the fabric colored with dyes **7** and **10** was found to be most affected by pH, according to figures (2–7) and Tables 2, 3 and 4. Furthermore, every figure and table showed that the synthetic dye number **7** was the most effective at dyeing PET fabric.

### Characterization of dyed polyester

Table 5 displays the fastness properties of polyester fabrics dyed with synthetic dispersion dyes under specified conditions (130 °C dyeing temperature, 4% shadow, 30-minute dyeing time). Among the fastness characteristics evaluated are light, washing, and crocking fastness. Light Fastness: The primary elements influencing the lightfastness of dyed polyester fibers are the type of substituent groups on the dye structures and the concentration of dye molecules in the fiber. The electron density surrounding the dye molecule can be altered by these substituents, which may have an impact on how the dye interacts with the fiber. After reduction clearing, the dyed samples exihibited excellent light fastness, with a ratings of 7 on the blue wool scale). The dyed samples’ washing fastness ratings ranged from acceptable to good (3/5). Dyes **7** and **10** demonstrated excellent light fastness (7) due to the addition of a 6-bromo-3-(2-hydrazino-thiazol-4-yl)-chromen-2-one (**6**) and hydrazonoyl halides (**9**) groups linked to a 3-acetyl-6-bromo-2 H-chromen-2-one (**3**) nucleus, which increases the dye’s molecular weight and photostability. All things considered, the generated dyes showed a high degree of photofading resistance^30^. Crocking Fastness: The results ranged from very good (for dye **7**) to outstanding (for dye **10**), with values ranging from 4 to 5/5. Again, the structural properties of dyes **7** and **10**, which raise molecular weight and encourage fixation to the polyester substrate, reduce color transfer during rubbing and are responsible for their improved fastness.

**Table 5:** Properties of dyeing polyester fabrics.

Dye number	Light fastness	Washing fastness	Crocking Fastness
**dye 1**	**7**	**3**	**4**
**dye 2**	**7**	**3**	**5**

## Conclusion

Two novel disperse dyes have been developed, and their chemical composition verified. The polyester fabrics were then colored using these dyes at various pH values (2, 4, 6, 8, and 10), temperatures (110, 120, and 130 °C), and durations (30 to 90 min). Dye **10**’s red-shifted and widened absorption bands demonstrate improved conjugation and charge-transfer properties. The optical characteristics are strongly influenced by the polarity of the solvent; for dye **10**, acetone promotes a higher apparent absorption efficiency than dye 7. Dye **10** is more colorful than Dye **7**. To achieve exceptional color strength in value (20.8), the optimal dyeing conditions for innovative dye **7** were 30 min, pH = 4, temperature 130 °C, and shade 5%. To produce outstanding color strength in values (21.7), the best dyeing conditions for innovative eco-friendly dye **10** were 30 min, pH = 2, temperature 130 °C, and shade 5%. Most importantly, the material describes how to use the proper dyeing conditions for the previously mentioned distribution dyes to dye polyester clothing with the best color effects.

Future work will focus on evaluating additional functional properties of the dyed fabrics, including antimicrobial and UV-protective activities, and extending their application to other fibers such as nylon and microfiber. Additionally, investigating other natural fruit extracts as catalysts and conducting economic feasibility studies will be essential to transition this green laboratory-scale protocol into large-scale industrial textile production.

## Electronic Supplementary Material

Below is the link to the electronic supplementary material.

Supplementary Material 1

## Data Availability

All data generated or analyzed during this study are included in this published article and its supplementary information file.
