# Supplementary material for: Eco-friendly synthesis of coumarins using lemon juice as a natural catalyst and application as disperse dyes on polyester fabric
Source: Sci Rep. 2026 May 26;16:16291. doi: 10.1038/s41598-026-53238-4 (PMC13213002; doi:10.1038/s41598-026-53238-4)
Supplement: Supplementary file 1 — Supplementary Material 1 [file 41598_2026_53238_MOESM1_ESM.docx]

**Eco-friendly synthesis of coumarins using lemon juice as a natural catalyst and application as disperse dyes on polyester fabric**

**Ali A. Ali^1^, Sawsan A. Fouad^2^ and Anhar Abdel‑Aziem^2^***

**^1^**Department of Chemistry, Faculty of Science (boys), Al-Azhar University, 11884 Nasr City, Cairo-Egypt

^2^Chemistry Department, Faculty of science(Girls), Al-Azhar University, Nasr City, Cairo, 11754, Egypt.

^*^Corresponding author: Anhar Abdel-Aziem

Email: [anhar@azhar.edu.eg](mailto:anhar@azhar.edu.eg)

<http://orcid.org/0000-0002-3871-3183>. Phone no.: +201112446998


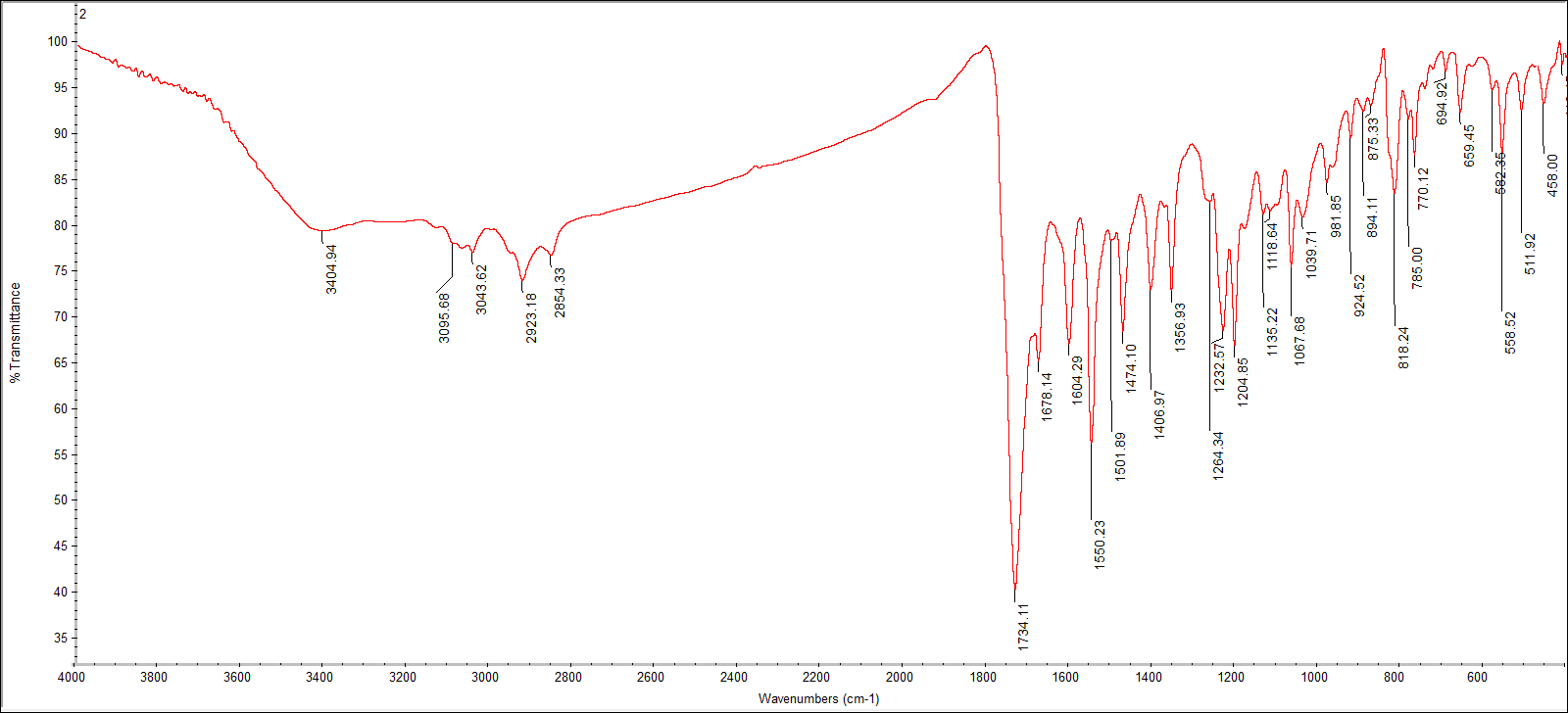
 **Figure s1.** IR spectrum of compound **7**


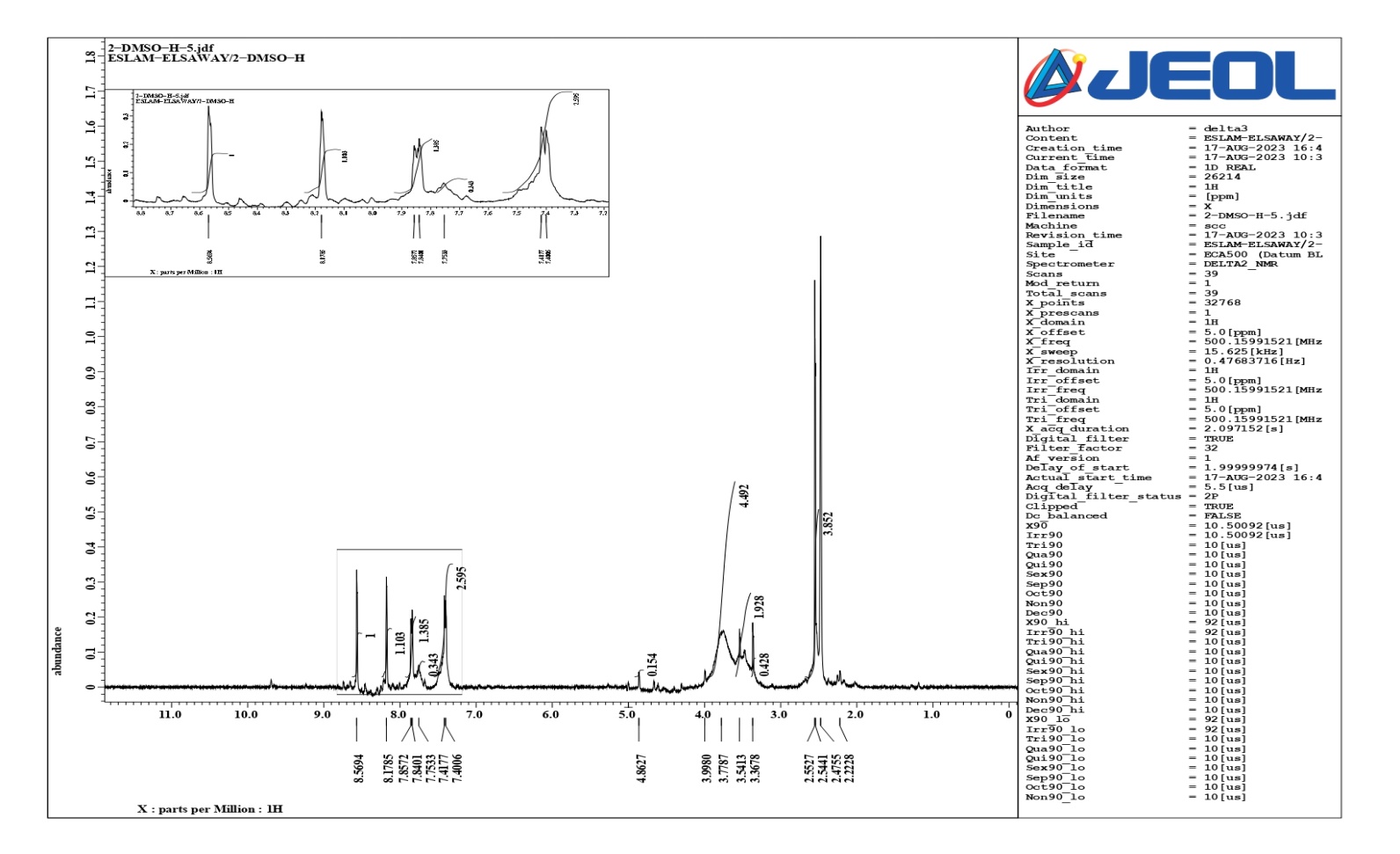

**Figure s2.** ^1^H NMR spectrum of compound **7**


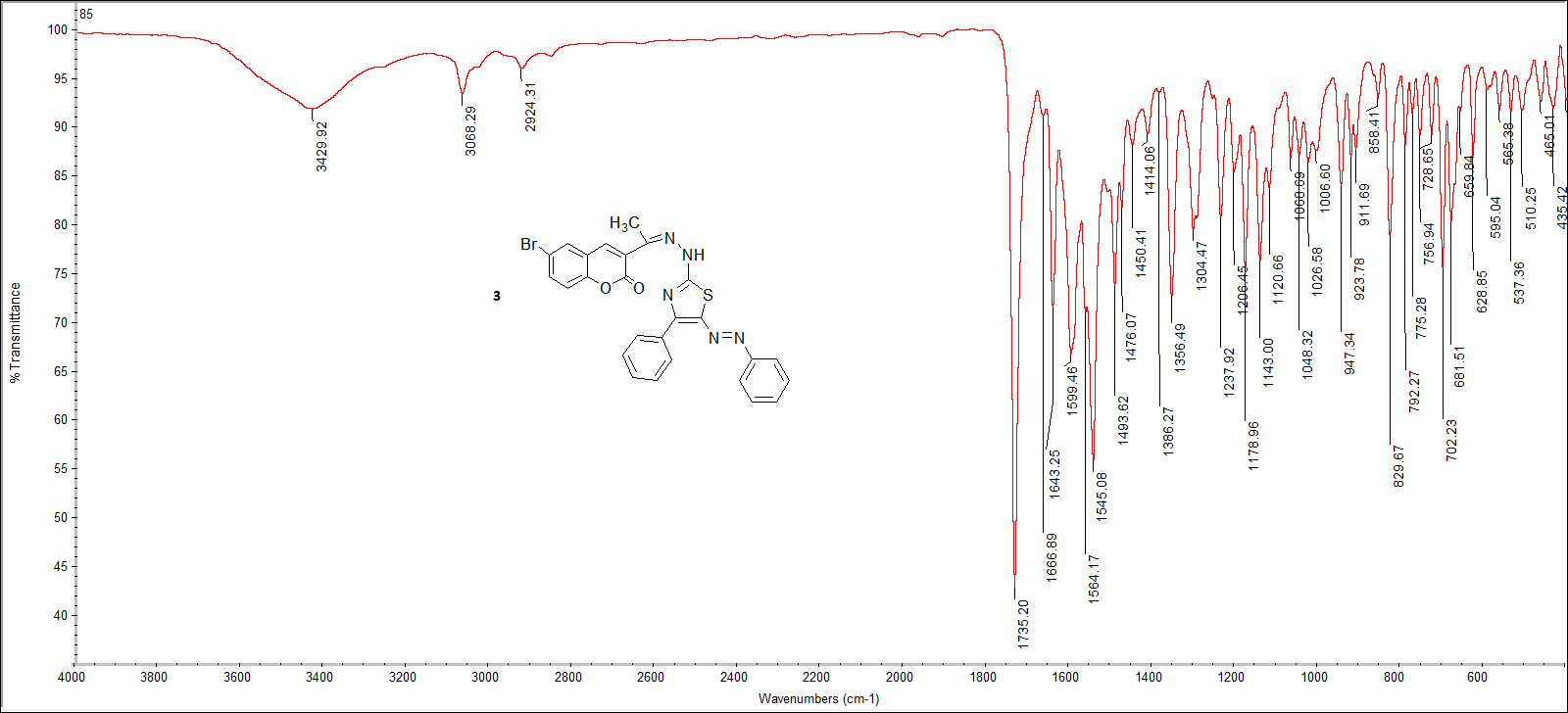


**Figure s3.** IR spectrum of compound 10


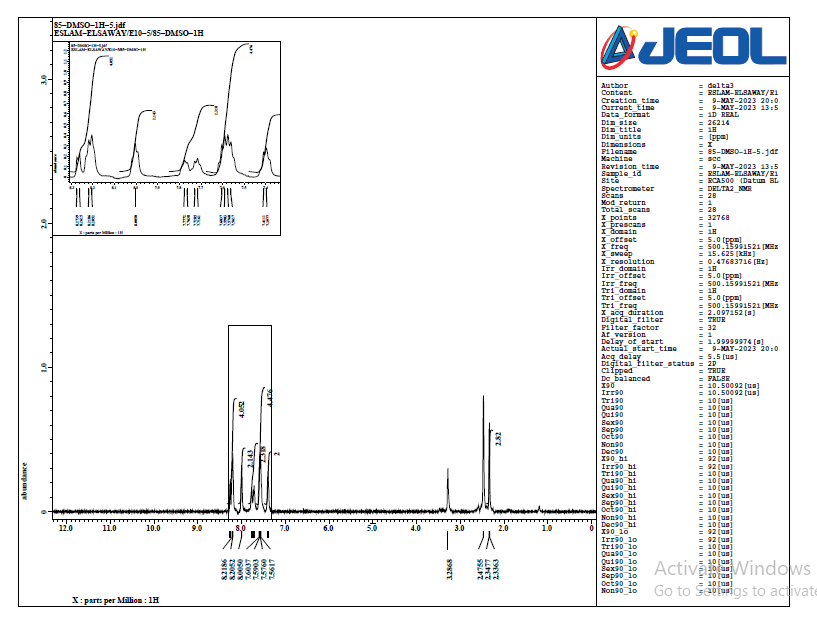

**Figure s4.** ^1^H NMR spectrum of compound 10


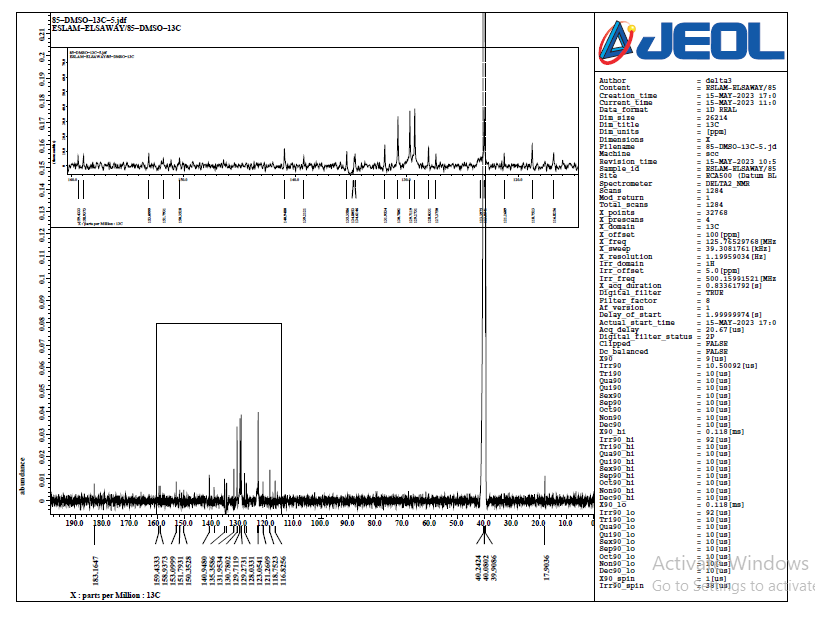

**Figure s5.** ^13^C NMR spectrum of compound 10
